# Extrinsic factors, endocrine mechanisms, and behavioral indicators of migratory restlessness in wintering whooper swans (*Cygnus cygnus*)

**DOI:** 10.1038/s41598-021-92031-3

**Published:** 2021-06-16

**Authors:** Liangliang Yang, Wenxia Wang, Ping Sun, Songlin Huang, Ruyi Gao, Desheng Kong, Wendong Ru, Torsten Wronski, Guogang Zhang

**Affiliations:** 1 Research Institute of Forest Ecology, Environment and Protection, Key Laboratory of Forest Protection of National Forestry and Grassland Administration, Chinese Academy of Forestry, Beijing, 100091 China; 2 Research Institute of Forestry Policy and Information, Chinese Academy of Forestry, Beijing, 100091 China; 3 Faculty of Forest and Environment, Eberswalde University for Sustainable Development, 16225 Eberswalde, Germany; 4 National Urban Wetland Park Administration, Sanmenxia, Henan China; 5 Faculty of Science, School of Biological and Environmental Sciences, Liverpool John Moores University, Liverpool, L3 3AF UK

**Keywords:** Zoology, Animal physiology

## Abstract

Extrinsic factors, endocrine mechanisms, and behavioral indicators of migratory restlessness were studied in wintering whooper swans (*Cygnus cygnus*) in the Sanmenxia Swan National Wetland Park in western Henan Province, central China. First, the fecal glucocorticoid metabolite (FGM) concentration was established and related to mean air temperature or photo period (day length) using simple linear or non-linear regression models. After a model selection procedure, the best fitted model revealed that an increase of FGM concentration was associated with an increase in the squared mean air temperature (R^2^ = 0.88). Other models showed an increasing FGM concentration to correspond with increasing values of day length, squared day length, and mean air temperature—however without statistical support. In a second step, behavioral frequencies of seven behaviors were condensed into three behavioral principal components (PCs) using principal components analysis. Behavioral PCs largely corresponded to three activity phases described for wintering whooper swans in central China and were correlated with the FGM concentration using Spearman's rank-order correlations. Results revealed a significant correlation between FGM and behavioral PC2 (positive factor loadings from vigilance and preening, negative loading from foraging). Finally, we tested for an effect of behavioral PCs on changes in winter home range size using a set of multiple linear regression models. Results of averaged model parameter estimates showed only the behavioral PC3 (positive factor loadings from fighting and calling, negative loading from locomotion) had a marginal significant effect on home range size. Results confirmed findings of previous studies on migratory restlessness in whooper swans. However, due to the small sample size (N = 15 weeks) the effect of PC3 on home range size was weak and should be viewed with caution.

## Introduction

Bird migration is an adaptation facilitating species to exploit resources in seasonally favorable areas and to increase their reproductive success. The annual cycle of palae-arctic migrants corresponds with seasonal changes in the ecosystem, prompting reproduction, post-breeding molt, autumn migration, wintering, pre-breeding molt and spring migration^1,2^. Breeding and wintering ranges of migratory birds are often thousands of kilometers apart, so that optimal exploitation of seasonal resources requires precise processes to regulate the timing of different migratory stages^3^. Bird migration is regulated by either intrinsic factors like the endogenous rhythm (i.e., the innate biological clock) and genetically fixed, often population specific, endocrinological control mechanisms^4–8^, or by extrinsic factors such as air temperature, daylength (photo period) and food availability^9–11^. Photoperiodic control of spring migration was experimentally demonstrated for several bird species breeding in temperate or arctic latitudes^5,12–14^. Daylength was shown to influence endogenous factors, e.g., being a timer of the circadian rhythm or by controlling the circannual rhythm through the production of hormones^4,15,16^. For example, the innate circannual rhythm in migrant birds has developed as a response to seasonal food availability and is thus the main mechanisms controlling the onset of spring migration^17^. Changing weather conditions were also reported to influence the migration of palae-arctic migrants. Spring migration is mainly related to increasing temperatures, while autumn departure corresponds to decreasing air temperature in the breeding range^18–22^. Such behavioral responses correspond to changes in the corticosterone level to meet physiological needs in the early stage of migration, such as an increase in foraging frequency to enhance the body fat content (refueling) for the upcoming spring migration^3,23^. Changes in day length and/or temperature can also cause a homeostatic imbalance in migratory birds, generating a series of physiological and behavioral responses to restore homeostasis (stress responses)^7,24,25^. Landys et al. have shown that the adrenocortical hormone level is tightly linked to migratory restlessness in birds (Zugunruhe^26^) and that a sufficient degree of migratory restlessness promotes migratory behavior^27–29^. Current studies on migratory restlessness and related corticosterone levels are often focused on the physiological responses and behavioral characteristics of birds during migration, their energy supplementation at stopover, the indicative functions of physiological responses or the influence of specific environmental factors^30–33^. Only a few studies were centered on the interface between environment, physiology, and behavior, limiting our understanding of the mechanisms underlying the adrenocortical responses and their regulation in migratory birds^34,35^.

The whooper swan (*Cygnus cygnus*), a class II protected bird species in China^36,37^, and is a circum-arctic migrant, breeding across the arctic tundra of North America, Europe and Asia. Populations using the East Asian Bird Flyway breed in the desert zone wetlands of Mongolia and the Russian tundra^38^, but spend the winter in the wetlands and coastal areas of central and eastern China (e.g., Sanmenxia in Henan Province, Rongcheng and Dongying in Shandong Province, Qinghai Lake and Korla in Xinjiang Province)^36,37,39^. The Sanmenxia wetland is among the most important wintering grounds of whooper swans in China^37^ and represents an ideal area to study adrenocortical responses to environmental changes such as temperature or photo period. The wintering swan populations in the Sanmenxia wetland is numerous and clumped in a relatively small area, allowing for efficient fecal sample collection, an adequate number of behavioral observations and the capture and radio-tagging of focal individuals. Due to detrimental effects on the timing of migration, earlier studies identified migratory *Cygnus* species as particularly sensitive to the effects of climate change^40,41^. To conserve whooper swan populations in the future, it is imperative to better understand the underlying control mechanisms of migration timing and migratory restlessness.

In our study we therefore explored the effects of two extrinsic factors on the endocrine metabolism and behavior of whooper swans prior to spring migration. In a first step we tested whether changes in ambient temperature and day length affected the glucocorticoid concentration of whooper swans. In a second step we examined whether the glucocorticoid concentration is related to changes in the activity budget, i.e., seven behavior variables, while in a third step we tested if such behavioral responses corresponded to changes in the home range size of whooper swans during the wintering period in the Sanmenxia Swan National Wetland Park. We predicted that whooper swans would exhibit physiological responses, i.e., an increase of stress hormones (fecal glucocorticoid metabolite, FGM concentration), following temperature and day length changes throughout the wintering period. We further hypothesized that migratory restlessness will be expressed by changes in the frequency of specific behaviors and an increase of the activity range towards the end of the wintering season. We used focal animal sampling and instantaneous scans, satellite tracking, and enzyme-linked immunosorbent assay (ELISA) to analyze the interactions between extrinsic factors, physiological stress responses and behavior changes of wintering whooper swans.

## Results

**Figure 1:**
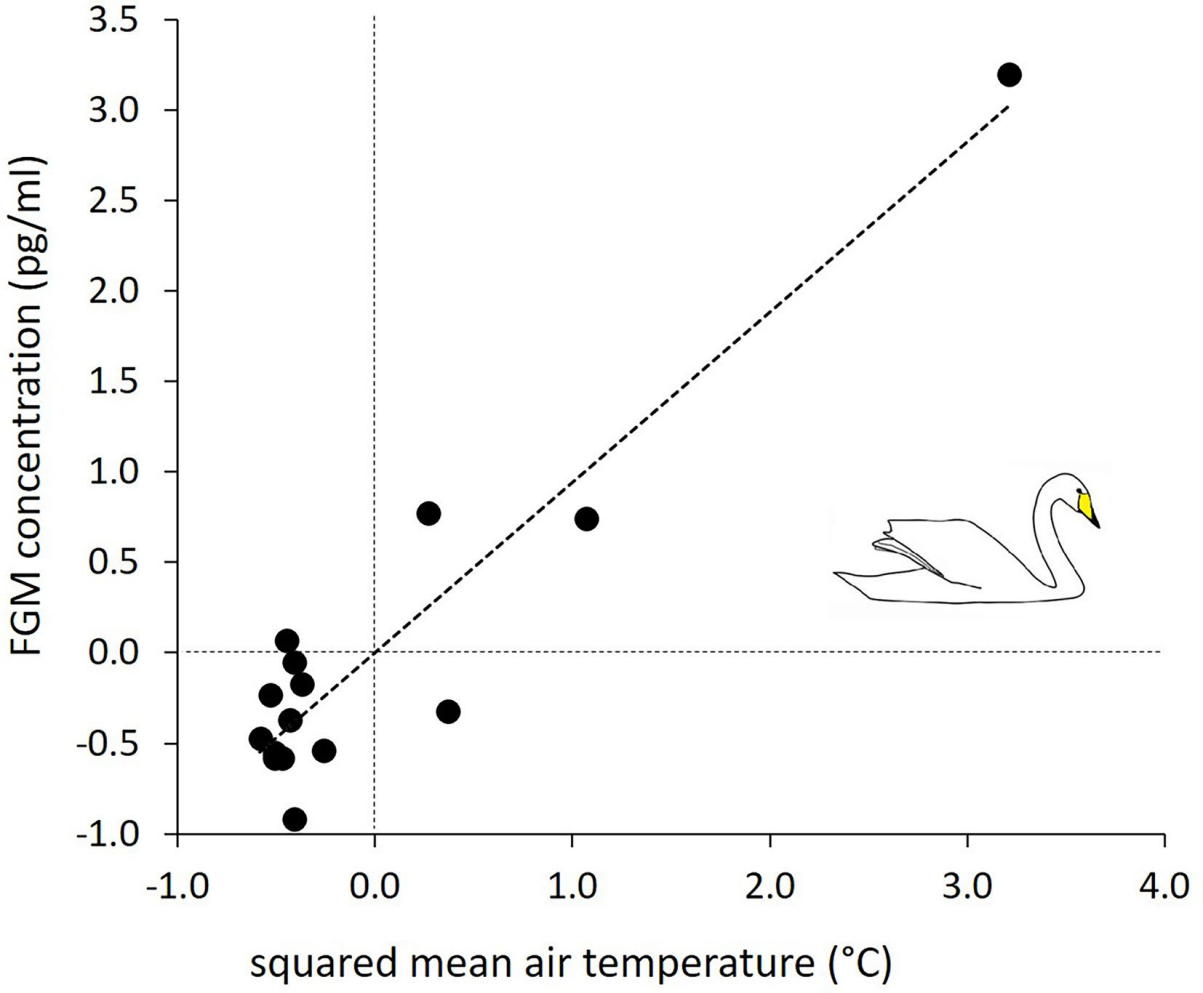
Best fitted non-linear regression model (Table 1), showing the relationship between FGM concentration, and squared mean air temperature (both z-transformed; R^2^ = 0.88).

**Figure 2:**
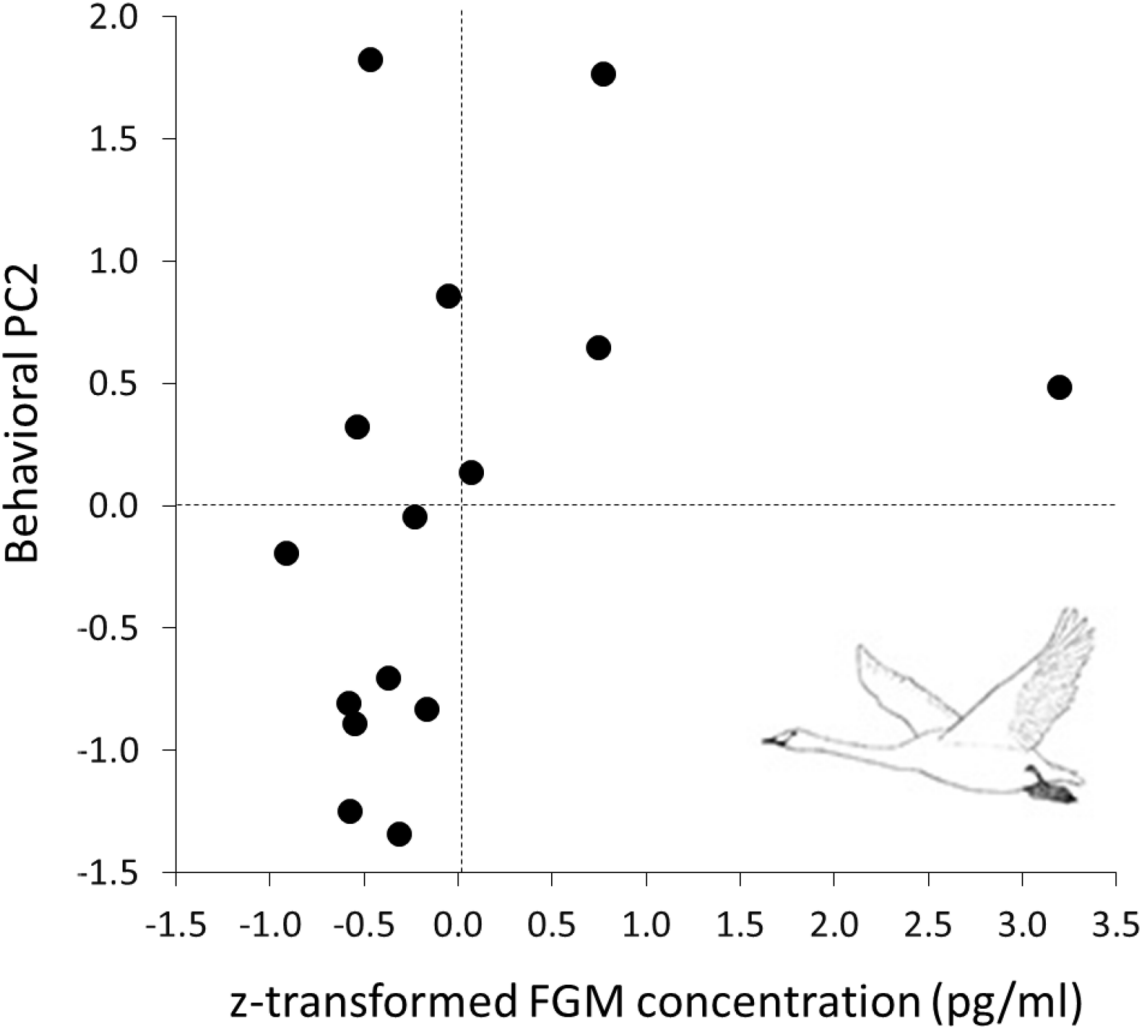
Relation (Spearman's rank-order correlation) between the behavioral PC2 and the fecal glucocorticoid metabolite (FGM) concentration.

A non-linear regression model, with the quadratic term of mean air temperature as the predictor, was selected as the best fitted model to unravel how FGM concentration was affected by extrinsic factors (Table 1). In this model, a significant increase of FGM concentration was associated with an increased squared mean air temperature (*R*^2^ = 0.88, Fig. 1). The principal component analysis revealed three behavioral PCs with an eigenvalue > 1.0, which explained 81.75% of the total variance (Table 2). PC1 received high positive factor loading from resting, and negative factor loadings from vigilance and preening (Table 2). These behaviors are characteristic for the inactive phase of the swan’s wintering period during which the swans show high frequencies of resting behavior but low frequencies of preening and vigilance behavior. PC2 received high positive factor loadings from preening and vigilance, but a negative loading from foraging (Table 2). This combination of behaviors is typical for the late, active phase of wintering swans during which they show migratory restlessness with high frequencies of alertness (vigilance) and preening, but low frequencies of foraging. PC3 received high positive factor loadings from fighting and calling, but negative loading from locomotion (Table 2), being characteristic for the middle phase of the wintering period during which the entire population has arrived in the wintering area and swans compete for food resources, try to keep contact to their kin through contact calls, while at the same time being sedentary with very low frequencies of locomotion inside the wintering range. Spearman rank-order correlations between the FGM concentration and PC1 revealed no significant relationship (*r* = − 0.34, *N* = 15, *P* = 0.22), while that between FGM and PC2 was significantly correlated (*r* = 0.52, *N* = 15, *P* = 0.05; Fig. 2). Moreover, no significant relationship was observed between PC3 and the FGM concentration (*r* = 0.01, *N* = 15, *P* = 0.96) as well as between home range size and the FGM concentration (*r* = 0.15, *N* = 15, *P* = 0.59). Furthermore, linear regression models revealed that none of the three behavioral PC combinations affected the home range size (Table 3). The null model was as plausible as the model including only PC3, or that comprising of PC1 and PC3 as predictors. Averaged model parameter estimates showed that PC3 had a marginal significant effect on the home range size in the wintering range (Table 4).

**Table 1:** Model selection summary of six candidate regression models.

Model	*k*	*logLik*	AICc	∆AICc	*w*_*i*_	*R*^2^
FGM ~ temperature^2^	3	− 47.61	103.4	0.00	0.85	0.88
FGM ~ temperature + temperature^2^	4	− 47.48	107.0	3.57	0.14	0.88
FGM ~ daylength + daylength^2^	4	− 51.29	114.6	11.18	0.00	0.81
FGM ~ temperature	3	− 54.10	116.4	12.98	0.00	0.72
FGM ~ daylength^2^	3	− 56.12	120.4	17.02	0.00	0.63
FGM ~ daylength	3	− 56.62	121.4	18.02	0.00	0.61
Null	2	− 63.60	132.2	28.79	0.00	–

The fecal glucocorticoid metabolite (FGM) concentration of wintering whooper swans was used as the dependent variable, while day length, mean air temperature as well as their quadratic terms were used as independent variables. Models were ranked by Akaike Information Criterion values corrected for small sample size (AICc) and weights (*w*_i_), whereby higher weighted models received more support. *K* number of parameters included in the model, *R*^2^ regression coefficient.

**Table 2:** Results of principal component analysis of seven behavioral variables obtained from 150 whooper swans.

Variable	PC1	PC2	PC3
Eigenvalue	2.00	1.91	1.81
% of variance	28.55	27.33	25.88
Resting	**0.89**	0.04	0.26
Vigilance	**− 0.81**	**0.51**	0.06
Foraging	− 0.02	**− 0.98**	0.14
Preening	**− 0.54**	**0.76**	0.16
Locomotion	0.28	0.11	**− 0.85**
Fighting	0.35	0.32	**0.71**
Calling	0.23	− 0.10	**0.69**

PC loadings >|0.50| are shown in bold font type.

**Table 3:** Model selection summary of seven hierarchical linear regressions ranked by Akaike information criterion values corrected for small sample size (AICc) from the lowest to the highest value.

Model	*k*	*logLik*	AICc	∆AICc	*w*_i_	*R*^2^
HRsize ~ PC3	3	− 18.79	45.80	0.00	0.34	0.23
Null	2	− 20.77	46.50	0.78	0.23	–
HRsize ~ PC1 + PC3	4	− 17.72	47.40	1.68	0.15	0.33
HRsize ~ PC1	3	− 19.96	48.10	2.35	0.11	0.10
HRsize ~ PC2 + PC3	4	− 18.43	48.90	3.10	0.07	0.27
HRsize ~ PC2	3	− 20.49	49.20	3.41	0.06	0.04
HRsize ~ PC1 + PC2 + PC3	5	− 17.30	51.30	5.52	0.02	0.37
HRsize ~ PC1 + PC2	4	− 19.65	51.30	5.55	0.02	0.14

Home range size (HRsize) of wintering whooper swans was used as the dependent variable, while the three behavioural PCs were used as predictors. *K* number of parameters included in the model, *w*_i_ model weight, *R*^2^ regression coefficient.

**Table 4:** Averaged model parameter estimates for the top three ranked linear models in Table 3.

	Estimate	SE	*Z*	*P*	2.5–97.5% CI
Intercept	0.00	0.24	0.00	0.99	− 0.52 to 0.52
PC3	− 0.48	0.24	1.81	0.07	− 1.00 to 0.04
PC1	0.32	0.24	1.22	0.22	− 0.19 to 0.83

## Discussion

To unravel how the corticoid concentration of wintering whooper swans was affected by extrinsic factors (photo period, ambient temperature), a set of hierarchical regression models was generated. The best fitted model revealed that an increase of FGM concentration was associated with an increase in the squared mean air temperature. This result suggests that air temperature was the main factor activating the endocrine control of spring migration in wintering whooper swans. Numerous studies have reported on the effects of photo period^5,11–14^ or ambient temperature^42–47^ on the spring migration of palae-arctic migrants. Since both parameters are inevitably linked—even in our study we found a strong correlation of both factors—it was proposed that both environmental factors influence the departure from wintering areas by influencing the endocrine control mechanisms^48,49^. It was further argued that long-distance migrants—spending the winter under relatively stable weather conditions in the tropics—rely mainly on endogenous mechanisms to initiate migration, i.e., the circannual rhythm, whereby short-distance migrants—wintering under more erratic conditions within the Palearctic—make use of exogenous factors such as air temperature^42,43^. Here, temperatures are subject to rather strong annual fluctuations, and mild weather conditions may induce migrants to depart earlier, while cooler weather conditions may delay their departure. In our study we could show that temperature can indeed kick-off endocrinological responses in Palearctic short-distance migrants. This finding is of particular interest, when considering rising ambient temperatures due to climate change. In recent years, an increasing number of studies found that the timing of spring migration has significantly shifted, both in the Palearctic and Nearctic migrants^43,50,51^. For example, studies on Bewick’s swans (*Cygnus bewickii*)—a close relative of whooper swan—unraveled shifts in the species wintering range as well as phenological changes, indicating that the timing of spring migration was affected by climate change (i.e., higher temperatures in the wintering range)^11,39,40^. Moreover, those studies identified migratory *Cygnus* species as particularly sensitive to the effects of climate change, ranking whooper swans as a species that will be greatly impacted by global warming^52^_._ To conserve swan populations in the future, it is therefore imperative to better understand the underlying control mechanisms of migration timing and to identify detrimental effects on the phenology of this species^53^.

While the corticosterone level depends largely on seasonal changes of endogenous factors, the hormone is also held responsible for triggering changes in the behavior of migrant bird species. In our second approach we used a principal component analysis to condense seven behavior variables into three behavioral PCs. Interestingly, the PCs defined in our study corresponded to three activity phases described for wintering whooper swans in central China^37,38,54^. The behavioral PC1 described the inactive phase of the swan’s wintering period during which the birds showed high frequencies of resting behavior but low frequencies of preening or vigilance (Table 2). This behavior component corresponded to the early wintering period (from late October to early January^54^), when temperatures were still above zero (average 3–15 °C) and wintering whooper swans arrived at the wetland. The behavioral PC3 was characterized by increased frequencies of fighting and calling, but also by a low degree of locomotion (Table 2). This combination of behaviors corresponded to the middle phase of the wintering period (from early January to early February^54^) when temperatures fell below zero (average − 5 to 8 °C) and the lake surface was covered by a solid layer of ice. During this period, the entire swan population had arrived in the wintering area and swans became very sedentary with small home ranges and only few movements within the study area. At this time swans were competing for limited food resources, while at the same time keeping close contact to their kin through contact calls. Behavioral PC2 described the late, active phase of the wintering period, during which the swans showed increasingly migratory restlessness, typically accompanied by high frequencies of alertness (vigilance) and preening^55,40^ (Table 2). By then swans had accumulated already enough fat reserves and therefore showed low frequencies of foraging. Moreover, migratory swans shrink the size of their guts, and hence a reduction in gut capacity immediately prior to spring migration could also contribute to the observed decrease in foraging behavior^56^. The late period reached from early February to the onset of spring migration in March^54^, and temperatures increased again to above zero (average 6–18 °C).

Given the perfect match of behavioral PCs and the seasonality of the study area, we asked whether this link was also reflected by a relationship between the FGM concentration and the behavioral PCs. We correlated the FGM concentration with three behavioral PCs and found a significant effect of FGM concentration on the behavioral PC2 (Fig. 2), suggesting that migratory restlessness was indeed driven by an increase of the adrenocortical hormone level. Several studies^28,57,58^ have described changing corticosterone levels to be indicative for the initiation of bird migration. Eikenaar et al. showed that bird migration was influenced by the combined effects of intrinsic, physiological factors and extrinsic, environmental factors confirming our finding that temperature changes and changes in day length were positively correlated to behavior changes and were thus indicative for the onset of migratory restlessness^3,31,33^. Once the corticosterone concentration increased beyond a certain threshold, swans were expected to leave their wintering areas and start their spring migration^3^.

Hormone-induced behavior changes (e.g., migratory restlessness) were also reported to be related to changes in the activity space, i.e., the home range size, of whooper swans in the wintering range^53^. In our study we found that the behavioral PC3 was marginally negatively related to home range size (Table 4), suggesting that decreased locomotion and high frequencies of fighting and calling were indicative for a reduced activity range during the mid-wintering phase. The behavioral PC3 was indicative for the middle phase of the swan’s wintering period, which was surprising, since we expected that an increased home range size at the end of the wintering period^54^ would correspond to increased frequencies of preening (pre-breeding molt), increased vigilance and lowered frequencies of foraging (i.e., behavioral PC2). However, this was not the case. Instead, the behavioral PC2, characteristic for the late, pre-migratory phase, revealed no significant effect on the home range size (Table 3). Previous studies have reported an increase of wintering home range size in migrant whooper swans towards the end of the wintering period, and that this increase corresponded to an increased frequency of certain behaviors^54^ (e.g., resting, foraging, and locomotion). Since only the behavioral PC2 was influenced by the FGM concentration but not PC3 (which marginally affected home range size), our results suggested no direct relation between the corticosterone level and the home range size. This result was further supported by an insignificant correlation between FGM concentration and home range size. However, the linear regression model suggested a rather weak effect of PC3 on home range size (Tables 3, 4). This might be attributed to the overall small sample size in our study (N = 15 weeks), which was unavoidable due to the irregular sampling of the various parameters (especially home range size) included in our analyses. In summary, our study confirmed a strong effect of extrinsic factors on the corticosterone concentration which in turn significantly correlated with the behavioral PC2 (a set of behaviors characteristic for migratory restlessness during the late wintering period). The effect of PC3 on the home range size, however, was weak and should be viewed with caution.

## Methods

### Study area and period

The study was carried out in the Sanmenxia Swan National Wetland Park (34.36° N–34.50° N, 110.21° E–111.23° E), located at the southern shore of the Yellow River (Huang He) in western Henan Province, central China^37^. The area covers 590 ha at an altitude of about 320 m above sea level. In August 2002, the municipal government protected the area as the Swan Lake State Urban Wetland Park, providing suitable wintering habitat for about 3.000 swans. During the wintering period, whooper swans prefer shallow open waters that provide abundant food^59^ (mainly aquatic plants and their roots) and safety from predators^37^. A few waterbird species share the wetland area with the swans at this time of the year, including the Eurasian coot (*Fulica atra*), black stork (*Ciconia nigra)*, some anatids like Chinese merganser (*Mergus squamatus*) and White-fronted goose (*Anser albifrons*), gulls (Laridae), and several migrating waders (Charadriiformes). No other swan species occurs in the wetland park. This study encompassed one complete wintering period of whooper swans in the study area, i.e., from late October when birds gradually begin to arrive, until late March when swans leave after completing the pre-breeding molt. Data collection and compilation of all variables included in our study (i.e., day length, mean daily temperature, FGM concentration, frequency of seven behavior variables and home range size) were based on weekly means, i.e., from week 46 in November 2017 to week 13 in March 2018. Due to poor weather conditions and the inaccessibility of the study area (i.e., persistent rain leading to higher water levels), week 4–5 and 8–10 are missing in our data set, resulting in a total of 15 sampling events across the study period.

### Extrinsic factors

Day length data were obtained from https://richurimo.bmcx.com, while mean daily air temperatures were obtained from http://tianqi.2345.com. To customize the extrinsic factors to the weekly sampling scheme, daily data were pooled and averaged for each week (Table 5).

**Table 5:** Sample number, week of the year, year and the extrinsic and intrinsic variables included in the weekly sampling scheme of wintering whooper swans in Sanmenxia Swan National Wetland Park (daily data were pooled and averaged for each week): photo period (day length in minutes), mean weekly air temperature (°C), mean fecal glucocorticoid metabolite (FGM) concentration (pg/ml), home range (HR) size (km^2^) and the frequency of seven behaviors: vigilance (V), fighting (G), preening (P), resting (R), calling (C), locomotion (L) and foraging (F).

Sample no.	Week	Year	Day length	Mean. temp	Mean FGM	V	G	P	R	C	L	F	HR size
1	46	2017	619.0	9.50	28.41	0.08	0.00	0.07	0.09	0.02	0.31	0.42	1.69
2	47	2017	612.0	4.00	17.99	0.07	0.00	0.07	0.17	0.05	0.30	0.33	2.03
3	48	2017	603.0	5.50	24.58	0.08	0.00	0.12	0.22	0.04	0.22	0.31	2.04
4	49	2017	596.0	3.50	35.12	0.08	0.01	0.09	0.18	0.03	0.24	0.36	0.95
5	50	2017	551.0	2.25	29.89	0.06	0.00	0.10	0.26	0.04	0.21	0.34	0.39
6	51	2017	589.0	2.50	24.33	0.05	0.01	0.08	0.20	0.03	0.20	0.43	0.86
7	52	2017	590.0	3.25	23.79	0.10	0.00	0.09	0.17	0.04	0.19	0.40	0.95
8	1	2018	592.0	4.50	30.97	0.14	0.00	0.09	0.13	0.04	0.19	0.41	0.44
9	2	2018	599.0	− 2.50	23.91	0.12	0.00	0.14	0.08	0.04	0.17	0.45	0.28
10	3	2018	607.5	3.75	27.44	0.18	0.00	0.11	0.08	0.05	0.20	0.39	0.20
11	6	2018	642.0	− 0.25	25.80	0.14	0.01	0.19	0.12	0.05	0.25	0.24	0.55
12	7	2018	656.0	4.00	33.00	0.19	0.00	0.15	0.10	0.03	0.22	0.30	0.30
13	11	2018	716.0	12.50	46.80	0.21	0.00	0.16	0.05	0.02	0.27	0.29	1.71
14	12	2018	726.0	9.00	47.28	0.22	0.00	0.17	0.15	0.03	0.20	0.24	1.37
15	13	2018	741.0	19.00	89.44	0.17	0.01	0.13	0.13	0.04	0.19	0.32	2.22

### Behavioral observations

Behavioral observations were carried out at distances of 200–300 m using a spotting scope (Swarovski, Austria) with a magnification of 20 × 60. Observations were conducted following procedures described in^55,60^, using instantaneous sampling with a sampling interval of 10 min. Ten individuals were simultaneously observed (scan sampling) on two or three consecutive days for a period of ten hours, i.e., between 8:00 in the morning and 18:00 in the afternoon. Focal individuals were chosen arbitrarily and were not individually distinguishable. Observations were terminated if the focal animal went out of sight and the total observation time was recorded. Subsequently, another individual within the field of view was selected and the observation continued, coming to a total sampling period of 250 h per week (2.5 days × 10 h × 10 individuals). The total number of individuals sampled was 150 (15 weeks × 10 individuals). Based on descriptions by Dong et al. as well as our own preliminary observations, seven behaviors were defined (Table 6)^61^. Behavior frequencies were expressed as percentage proportions of the total observation time for each individual sampled and subsequently pooled and averaged for each week (Table 5).

**Table 6:** Definitions of seven behaviors observed in wintering whooper swans at Sanmenxia Wetland in western Henan Province, China^61^.

Behavior category	Behavior explanation
Vigilance	With a head-up posture, scanning or staring at the surrounding environment (looking at the source of strong interference); occasionally rotating the head
Fighting	Chasing, spreading wings, and biting
Preening	Using the beak to tidy up feathers around the body, flapping and spreading wings, rolling in water to bath, and cleaning the feet, etc.
Resting	Standing still or taking a rest. When resting, the head usually turns back, and the front end of the beak hides underneath the wings. Occasionally raising the head when alerted, then continuing to rest
Calling	Giving ‘hour’ or ‘ho-ho’ like calls
Locomotion	Including flying, swimming in water, and walking on land or ice
Foraging	Including foraging on land, filtering food with the beak on the water surface, and searching for food underwater

### Capture, radio tagging and home range size estimation

Between October 2017 and March 2018, 25 whooper swans were trapped at five locations within the Sanmenxia Swan National Wetland Park using cannon netting^62^. This method allowed us to take a random sample of the total swan population in the study area at different stages of their molt. After capture, swans were equipped with satellite transmitters (YH-GTG0325, Hangzhou Yuehai Technology Ltd., China) and immediately released into the wetland. Each devise weighed approximately 22–30 g, accounting for 0.2–0.3% of the average body weight of a whooper swan. Transmitters were equipped with internal GPS receivers, solar panels and external antennae, and were attached dorsally with a 1.4 cm wide woven tubular Teflon ribbon. Signals were received by the communication system of China Mobile, interpreted by the network client, and downloaded to a personal computer. Tracking data included time, latitude, longitude, and accuracy, whereby accuracy was measured as the position dilution of precision (PDOP). Radio-tagged individuals were tracked for different periods depending on the time they were captured. Only two individuals could be tracked for the complete wintering season, i.e., from early November 2017 to the end of March 2018. Sample sizes for monthly home range estimates were therefore: four individuals in November, six in December, 10 in January, 25 in February and 22 in March. A total of 9308 location fixes with a PDOP ≤ 2.0 (corresponding to an accuracy of ≤ 20 m) were obtained from radio transmitters. Individual location fixes were processed using the ‘adehabitatHR’ package in R (version 3.3), to calculate home range extensions as 90% fixed kernel density estimates^62,63^. Subsequently, Kernel home range estimates were pooled and averaged for each week (Table 5).

### Fecal sample collection

Fresh fecal samples were collected every five days from 16 November 2017 to 27 March 2018. In some cases, this was not possible due to harsh weather conditions and changing water levels in the wetland (see above). Ten sampling sites were identified based on the distribution of wintering swans in the study area and the accessibility of their terrestrial resting places. Three parallel transects were established at each sampling site, covering most parts of their terrestrial resting area. Transect length differed, depending on the topography of the area (i.e., 100–500 m). Transects were walked on foot, with one observer on each transect line. Six to 30 fecal samples were collected at random along each transect line, during each survey and stored in resealable plastic bags at − 80 °C until further processed in the laboratory.

### Extraction of steroid metabolites from fecal samples

Preserved fecal samples (0.5 g ± 10 mg) were weighed and transferred into a 15 ml centrifuge vial. Subsequently, 5 ml of 90% ethanol was added, and the tube was vortexed until the sample was completely dissolved in ethanol. Each sample was vortexed again for 10 s and centrifuged for 15 min at 4000 rpm, and then for 25 min at 1500 rpm. The supernatant was decanted and stored in a separate test tube. Another 5 ml of 90% ethanol was added to the test tube containing the original fecal sample, vortexed again for 30 s and centrifuged for 20 min at 1500 rpm. Subsequently, the supernatant was added to the test tube containing the supernatant of the first extraction and dried after a water bath at 70 °C. After drying, the test tube wall was rinsed with 90% ethanol (approximately 2 ml), vortexed for 5 s, and dried again. Samples were recovered using 1 ml of methanol, vortexed, suspended to ultrasonic dissolution, and dried under the fume hood. Finally, 1 ml of diluent (NaH_2_PO_4_; Na_2_HPO_4_; NaCl; Milli-Q H_2_O, pH 7.0) was added to each sample, again suspended to ultrasonic dissolution for 15 min, sealed, and stored at − 80 °C for further use. Finally, an ELISA test was applied to measure the ultimate FGM concentration for each sampled individual. Individual FGM concentration was later pooled and averaged for each week (Table 5).

### Statistical analysis

In all cases, measurements were pooled and averaged across weeks to obtain one value for each week. Prior to statistical analyses we arcsine (square root)-transformed all behavior frequencies. Subsequently, we applied z-transformation to the entire data set to obtain standardized values as variables (mean = 0, SD = 1).

In a first step, we generated a set of six candidate regression models to test whether extrinsic factors such as day length or mean air temperature had a significant effect on the FGM concentration (dependent variable). Since day length was significantly correlated to mean air temperature (Spearman rank-order correlation: *r* = 0.65, *N* = 15, *P* = 0.01), models were built to avoid collinearity. Our model predictors therefore included either day length or mean temperature, as well as their quadratic terms. A model selection process^64^ was carried out by ranking Akaike Information Criterion values corrected for small sample size (AICc) and model weight. The best fitted model was identified by a low AICc and a ∆AICc > 2.0 compared to the second lowest model. In cases where several top-ranked models showed strong similarities (∆AICc ≤ 2.0), a model averaging procedure was applied to obtain their mean.

In the second step we tested whether the FGM concentration was correlated to any of the seven behavior variables. Behavioral frequencies were condensed by applying a dimension reduction technique (i.e., principal components analysis, PCA) using the varimax rotation option. The resulting three principal components (PCs) with an eigenvalue > 1.0 (Table 2), were used to test for a relationship with the FGM concentration by employing three independent Spearman's rank-order correlation. Additionally, we correlated the FGM concentration with the home range size using a Spearman's rank-order Correlation. In a third step, we tested whether the same three behavioral PCs affected the home range size of whooper swans in the study area. We built a full set of hierarchical linear regression models including all possible combinations of behavioral PCs as independent variables, while home range size was specified as the dependent variable. A model selection (same as in the first step) was carried out to identify the best fitted, final model. All data analyses were carried out using R (version 3.5.1)^65^. The R packages ‘psych’ and ‘factoextra’^66,67^ were applied for PCA analysis, while the package ‘MuMIn’^68^ was used for model selection and averaging procedures.

### Permission statement

The research on wintering whooper swan carried out in this article has been permitted by Sanmenxia Swan National Wetland Park, and received a lot of support and help from the park to make the research work proceed smoothly.
